# Economic cost of Hansen’s disease: an overview of social security expenditures

**DOI:** 10.3389/fpubh.2026.1672733

**Published:** 2026-04-16

**Authors:** Jose Marcelo Castro, Filipe Rocha Lima, Marco Andrey Cipriani Frade, Claudia Souza Passador

**Affiliations:** 1 Department of Business Administration, School of Economics, Business Administration and Accounting at Ribeirão Preto, University of São Paulo, São Paulo, Brazil; 2 Division of Dermatology, Department of Internal Medicine, National Referral Center for Sanitary Dermatology and Hansen’s Disease, Ribeirão Preto Medical School, University of São Paulo, São Paulo, Brazil; 3 Department of Biochemistry and Immunology, Ribeirão Preto Medical School, University of São Paulo, São Paulo, Brazil

**Keywords:** burden of disease, cost of illness analysis, economic burden, leprosy, social security benefits

## Abstract

**Introduction:**

Brazil is the second highest worldwide regarding new cases of Hansen’s disease (HD), with over 20,000 diagnoses annually. Late detection often results in severe disabilities that impair daily functioning, reduce work capacity, and increase reliance on state financial assistance, which contribute to public expenditure. This study quantified the economic impact of Hansen’s disease by analyzing social security benefits in relation to the disease’s epidemiological profile from the late 20th century to the first two decades of the 21st century.

**Methods:**

This retrospective, longitudinal, descriptive study applied a cost-of-illness (COI) approach from a societal perspective. Indirect costs attributable to Hansen’s disease were estimated using social security transfer payments as proxies for productivity loss. Administrative data from the National Institute of Social Security were obtained for all benefits granted between 2000 and 2019. These records included temporary, long-term, and lifetime benefits, which were categorized by type, duration, sex, geographic location, and total financial disbursement. The economic evaluation considered both annual and accumulated payments over the study period. To contextualize the burden of disease, benefit data were analyzed alongside epidemiological indicators from the Ministry of Health’s Notifiable Diseases Information System, allowing the examination of the relationship between Hansen’s disease trends and variations in benefit concessions.

**Results:**

Systematic variations were identified in the number of benefits granted, with a marked increase until 2005, followed by an abrupt drop and a subsequent gradual decline. These fluctuations were influenced by exogenous monetary and political factors and changes in benefit requests. Long-term benefits followed the same pattern, stabilizing at approximately 1,000 grants per year. Temporary benefits showed recurrent oscillations and later declined from 4,283 in 2000 to 3,525 in 2019, remaining substantially more frequent than lifetime benefits. Data segmentation showed the following: (I) age at entry into the social security system peaked at 45 to 55 years for both sexes; (II) men received 2.7 times more benefits; (III) benefit duration was similar for men and women, with 52.0% ending within 18 months and 85.0% within 120 months; (IV) the Southeast region accounted for 21.8% of benefits and 24.8% of new cases; and (V) total payments increased over 60-fold, reaching US$ 91 million in 2019.

**Conclusion:**

Social security expenditures for beneficiaries with Hansen’s disease totaled US$ 924.6 million, representing a substantial economic burden relative to the disease’s epidemiological characteristics in Brazil. Although incidence has declined and benefit concessions have gradually decreased, partly due to improvements in administrative processes and Hansen’s disease control, the financial impact remains significant. Given that Hansen’s disease is curable, and treatment is freely available in the public health system, current resource allocation prioritizes compensation for productivity loss over early diagnosis and prevention. The identified disparities, including gender differences and concentration of benefits in wealthier regions, warrant further investigation to inform mitigation strategies.

## 1 Introduction

Since the mid-twentieth century, an expansion in the quality of life has been accompanied by a significant increase in human longevity. While this represents progress, its consequences have raised concerns regarding the sustainability of economic and social structures in many countries (1), especially those where social well-being depends heavily on public funding. Greater longevity requires more resources for pensions, and individuals affected by serious illnesses often need financial support during recovery or as compensation for resulting limitations (2). In Brazil, resources allocated to health promotion, treatment, and rehabilitation, as well as pensions and social benefits, are drawn from the Social Security System (3).

The combined effect of these expenditures has produced an upward spiral in social spending, prompting debates on social security reform. This debate concerns both the function of social protection and the criteria for granting pecuniary benefits. In Brazil, increased longevity has intensified the pressure to modify benefit rules, including raising the minimum retirement age, extending contribution time, reducing benefit amounts, and tightening eligibility for sickness benefits and other compensation funded by the social security system (4). Although motivations vary, most discussions focus on public finances and aim to reduce fiscal imbalances, thus making the system more balanced and sustainable (4). This reflects predominantly managerial and economic perspectives.

Another relevant dimension of national and international discussions is the extension of social protection to economically vulnerable groups and the potential impact of reforms on this protection. In this context, several aspects warrant attention, including the characterization of benefits granted for Hansen’s disease (HD) (5).

HD is a chronic infectious disease caused by *Mycobacterium leprae* and *Mycobacterium lepromatosis*. Transmission occurs through prolonged close contact with untreated individuals, mainly via inhalation of bacilli in the upper airway secretions. Signs and symptoms involve neurological and dermatological manifestations, and diagnosis is based on at least one of the following three cardinal signs: loss of sensation in a hypopigmented or reddish patch, thickened or enlarged peripheral nerves with sensory or motor impairment, or identification of bacilli in a clinical sample. Additionally, the diagnosis is primarily clinical, supported by epidemiological assessment, and complemented by auxiliary clinical and laboratory tests. The lack of early therapeutic intervention for neural impairment is the main cause of disabilities and deformities resulting from sensory loss in affected areas, ultimately compromising individuals’ work capacity and daily functioning (6).

Although HD is curable, it continues to cause physical disabilities, stigma, and, frequently, financial dependence on public support. Brazil is the second country with the highest number of cases worldwide, with more than 29,000 annual notifications until 2019 (7). Case detection sharply declined after the onset of COVID-19 because of disruptions in surveillance and care (8, 9). Globally, 182,815 new cases were detected in 2023, a 5% increase compared to 2022, and the number of grade-2 disabilities also increased (10, 11). In Brazil, although the incidence and prevalence have decreased (10), the proportion of multibacillary cases has increased from 64.4% in 2013 to 81.2% in 2022, and the proportion of new cases with grade-2 disabilities has risen from 7.3 to 11.5%. Currently, 44.3% of new cases present some degree of disability (10).

Considering the endemic profile of HD in Brazil, the availability of effective and free treatment, and the recent decline in notifications, a reduction in social security cost-of-illness might be expected. This study, therefore, quantifies the economic burden of HD on the Brazilian social security system between 2000 and 2019 by measuring the benefits paid to affected individuals. Aggregated and disaggregated analyses by benefit type, duration, geographic region, and sex were performed to support the interpretation and contextualize the national response to the disease.

Although this investigation applies economic methods in an epidemiological context, its broader aim is to highlight the need for health policies that are effective, efficient, and equitable health policies.

## 2 Materials and methods

### 2.1 Study design and data collection

To quantify the economic burden of HD on Brazil’s social security system, a partial economic evaluation using the COI approach was conducted, which measures the economic impact of a disease by evaluating the resources consumed and lost (12, 13). As a partial evaluation, it examines a single condition without comparing alternative interventions, thus serving as a descriptive cost framework (14, 15).

This study adopts a societal perspective—the broadest economic viewpoint—that incorporates all costs borne by individuals, governments, and health systems, regardless of who incurs or receives them (12, 14, 16). Cost estimation consists of aggregating the value of the social security benefits paid to individuals affected by HD. These transfers, including sickness allowances and disability pensions, represent indirect costs, as they reflect productivity losses resulting from the removal of workers from economic activity (14). Although formally transfer payments, COI studies commonly use their monetary value as a proxy for societal income and productivity losses caused by the disease (15). Thus, social security expenditures capture the externality of HD in the form of foregone productivity.

From a theoretical-operational standpoint, this longitudinal study has descriptive and exploratory characteristics. It analyzed the temporary and lifetime benefits granted by the Brazilian Social Security System due to HD, including their sums and percentages. The social security data analyzed covered all benefits granted between 2000 and 2019 and were provided by the National Institute of Social Security (Portuguese acronym: INSS), an agency of the Federal Government linked to the Ministry of Economy and responsible for managing resources and executing social security payments.

The data file was provided electronically by the INSS upon a formal request for this study and contained 121,738 records with the following variables: species code; benefit type (full name); gender; benefit amount in the current value on the date of the last payment; International Classification of Diseases (ICD-10); date of birth; benefit start date (BSD); benefit cessation date (BCD); cause of termination or suspension; municipality (with coding, state acronym, and name); and benefit status, classified as “Active” for ongoing benefits, “Terminated” for definitively discontinued benefits, or “Suspended” for benefits with interrupted payment due to beneficiary non-compliance (such as absence from medical examination). For all records, 31 December 2019 was assumed to be the cutoff date.

### 2.2 Study population and descriptors

Beneficiaries who received social security benefits between 2000 and 2019 as a result of being treated for HD or experiencing sequelae caused by the disease were included in the analysis. The selection of records of interest was made according to the ICD-10, comprising only the values for the variable ICD-10, which included A30 Leprosy; A30.0 Indeterminate Leprosy; A30.1 Tuberculoid Leprosy; A30.2 Borderline Tuberculoid Leprosy; A30.3 Borderline Leprosy; A30.4 Borderline Lepromatous Leprosy; A30.5 Lepromatous Leprosy; A30.8 Other Forms of Leprosy; A30.9 Unspecified Leprosy; and B92 Leprosy Sequelae.

### 2.3 Analysis strategy

This analysis was performed using secondary, anonymized data retrieved from publicly accessible national databases upon request. The database was filtered to include only benefits with start and end dates within the study period. This analysis adopts a human capital approach and uses Social Security benefit payments as proxies for indirect costs to quantify the fiscal burden borne by the Social Security system.

To characterize the benefits, the data were aggregated by species, sex, and place (federal units) and quantified in absolute and relative values. Owing to the variable characteristics over time and the fact that only the amount of the last payment was provided, specific processing was required to recompose the monthly payments retroactively.

Based on the data provided by the INSS, for beneficiaries whose last registered payment in the system corresponded to the current minimum wage, it was considered that, since the benefit was granted, the reference value had remained constant. In other words, it was assumed that the beneficiary received the minimum monthly wage throughout the concession period.

Based on this premise, the value of the minimum wage in effect for each month was assigned to the benefit, taking into account the interval between the monthly payment date and the previous annual salary adjustment. It should be noted that in Brazil, the minimum wage is defined annually by the Federal Government through a specific Federal Law.

In cases where the last amount paid to the beneficiary exceeded the minimum wage in force on the date of payment, the amounts were corrected using annual adjustment tables established by complementary legislation applicable to benefits that exceed this amount. As with the group receiving the minimum level, the last payment reported by the INSS was used to retroactively recompose the monthly amounts, covering the period up to the previous year’s adjustment date, and subsequently, the benefit start date.

Definitions:

For each beneficiary $i\in \left\{1,2,\ldots ,N\right\}$ where N is the total number of records:

• $B_{i}\left(t\right):$ benefit amount for beneficiary i in month t.

• $B_{i}^{last}:$ last nominal payment recorded for the beneficiary i

• MW(t): minimum wage in effect during the month t

• A(t) : adjustment factor applicable in the month t , based on complementary legislation

• $T_{i}=\left\{t_{0},t_{1},\ldots ,t_{last}\right\}:$ set of months from the start of the benefit ( $t_{0}$ ) to the last recorded payment month ( $t_{last}$ )

$$B_{i}\left(t\right)=\left\{\begin{array}{c} MW\left(t\right),\;\;\;if\;B_{i}^{last}\leq MW\left(t_{last}\right)\\ B_{i}^{last}\cdot \prod_{k=t}^{t_{last}}\frac{1}{A\left(k\right)},if\;\;\;B_{i}^{last}\;\;>\;\;MW\left(t_{last}\right)\;\; \end{array}\right\}\mathrm{for}\;\mathrm{all}\;t\in T_{i}$$

This procedure allowed the reverse attribution of monthly amounts using the last installment paid as the reference and progressively adjusting the values back to the beginning of the benefit period. After obtaining the nominal values of each monthly payment for all beneficiaries included in the analysis, monetary adjustment was performed using the National Consumer Price Index (Portuguese acronym: INPC) for December 2019 as the reference period (8, 17).

- Monetary update to December 2019

Let *I(t)* be the cumulative INPC index from month *t* to December 2019:


$$B_{i}^{real}\left(t\right)=B_{i}\left(t\right)\cdot I\left(t\right)$$

This update is necessary because comparing monetary values over relatively long periods requires that the amounts originally expressed in local currency be standardized to a single temporal reference. Thus, it is possible to ensure equivalence between the values over time, considering the effects of accumulated inflation throughout the period analyzed (18).

The monetary update, therefore, ensures comparability between monthly payments made at different times, allowing a methodologically consistent analysis adjusted for variation in the purchasing power of the currency. For international comparability, the final annual amounts already corrected and deflated were converted into US$ using the December 2019 exchange rate as the reference.

- Conversion to US$:

Let $E_{USD}$ be the exchange rate (BRL/USD) in December 2019:

$$B_{i}^{\mathrm{USD}}\left(t\right)=\frac{B_{i}^{\mathrm{real}}\left(t\right)}{E_{\mathrm{USD}}}$$

- Annual total per beneficiary (in USD):
$$Total_{i}=\underset{t\in T_{i}}{\sum }B_{i}^{USD}\left(t\right)$$
- Aggregates across all beneficiaries:
$$GrandTotal=\overset{N}{\underset{i=1}{\sum }}Total_{i}$$

Regarding the epidemiological data, the following indicators were used: number of new cases detected, percentages of disability assessment, and patients with Functional Disability Grade 2 (FDG2), obtained from the Notifiable Diseases Information System (Portuguese acronym: SINAN) of the Ministry of Health (19–21), which were organized in annual sequence tables. To calculate the relative percentage of FDG2, the probability of evaluation of all new cases in the year was considered, based on the observed percentage of effective evaluations and the number of new cases with FDG2 in that year.

Scientific literature, legislation, and reports were used to support the understanding and justification of the social, economic, political, and epidemiological phenomena observed at different levels of data aggregation of these data. All procedures for processing the database in this study were performed using Microsoft Excel 365, and graphs and tables were generated. The maps were generated in QGIS version 3.24 to illustrate and summarize the analyzed information.

## 3 Results

### 3.1 Epidemiological indicators of HD in Brazil from 2000 to 2019

From January 1, 2000, to December 31, 2019, a total of 752,528 new cases of HD were diagnosed in Brazil (Table 1), according to SINAN (19). During this period, 119,175 benefits were granted by Social Security (Table 2), corresponding to a coverage of 15.8% among individuals diagnosed. To present the cases of disease and the granting of benefits from a temporal perspective, the aggregate results are organized into two groups. The first refers to data on the occurrence of HD, intended to provide the epidemiological context over the study period. The second group covers the benefits granted, which are subdivided into topics with distinct characteristics identified as relevant.

**Table 1:** Epidemiological indicators of HD in Brazil from 2000 to 2019.

Year	New cases	General detection coefficient (per 100,000 inhab.)	Prevalence coefficient (per 10,000 inhab.)	Effective % FDG evaluation	Number new cases with FDG2	% of FDG2	Number new cases with relative^a^ FDG2	% Relative FDG2
2000	43,196	25.44	4.71	83.2	3,067	7.1	3,686	8.5
2001	45,874	26.61	3.99	84.7	2,753	6.0	3,250	7.1
2002	49,438	28.33	4.33	84.2	2,917	5.9	3,464	7.0
2003	51,900	29.37	4.52	84.9	2,906	5.6	3,423	6.6
2004	50,583	28.24	1.71	84.8	2,933	5.8	3,458	6.8
2005	49,464	26.86	1.48	85.5	2,868	5.8	3,354	6.8
2006	43,652	23.37	1.41	86.6	2,488	5.7	2,873	6.6
2007	40,126	21.19	2.11	83.0	3,772	9.4	4,544	11.3
2008	39,047	20.59	2.06	88.2	3,007	7.7	3,409	8.7
2009	37,610	19.64	1.99	89.3	2,708	7.2	3,032	8.1
2010	34,894	18.22	1.56	89.4	2,512	7.2	2,810	8.1
2011	33,955	17.65	1.54	89.5	2,411	7.1	2,694	7.9
2012	33,303	17.17	1.51	88.6	2,531	7.6	2,857	8.6
2013	31,044	15.44	1.42	88.1	2,266	7.3	2,572	8.3
2014	31,064	15.32	1.27	87.0	2,050	6.6	2,327	7.5
2015	28,761	14.07	1.01	87.1	2,157	7.5	2,474	8.6
2016	25,218	12.23	1.10	87.3	1,992	7.9	2,282	9.8
2017	26,875	12.94	1.35	87.1	2,231	8.3	2,561	9.5
2018	28,660	13.70	1.48	86.5	2,436	8.5	2,816	9.8
2019	27,864	13.23	1.50	85.6	2,351	8.4	2,747	9.9

a Estimates made by the authors, considering 100% of the new cases evaluated for FDG2.

Data Sources: (10, 19). FDG, Functional Disability Grade; inhab, inhabitants.

**Table 2:** Distribution of benefits granted by type of disease classification, 2000–2019.

Disease classification	2000	2001	2002	2003	2004	2005	2006	2007	2008	2009	2010	2011	2012	2013	2014	2015	2016	2017	2018	2019	Total
A30 - Leprosy	1,276	1,540	2,432	2,950	3,747	3,720	3,803	3,521	3,944	3,641	3,684	3,648	3,534	3,353	3,299	2,367	2,115	1,784	2,091	1,930	58,379
A30.0 - Indeterminate leprosy	276	271	297	277	238	184	130	124	118	137	127	113	105	86	113	75	88	72	79	67	2,977
A30.1 - Tuberculoid leprosy	158	170	191	162	178	208	208	166	143	127	110	106	95	80	69	56	46	36	43	31	2,383
A30.2 - Borderline Tuberculoid leprosy	33	37	54	44	51	64	61	40	40	45	32	47	43	29	32	26	23	20	19	11	751
A30.3 - Borderline leprosy	524	596	670	690	754	728	669	578	601	547	577	541	506	510	440	333	321	239	266	237	10,327
A30.4 - Borderline lepromatous leprosy	59	57	122	97	107	116	99	99	95	97	86	88	95	97	82	57	48	60	44	53	1,658
A30.5 - Lepromatous leprosy	565	551	771	689	670	600	523	479	497	479	490	465	442	354	364	279	242	215	213	166	9,054
A30.8 - Other forms of leprosy	165	154	142	125	83	68	57	37	32	30	45	45	44	43	36	34	32	27	31	31	1,261
A30.9 - Unspecified leprosy	650	662	847	674	745	655	570	345	383	410	471	425	409	351	351	271	275	201	222	166	9,083
B92 - Sequelae of leprosy	577	788	1,227	1,275	1,519	1,287	1,183	1,230	1,382	1,459	1,505	1,478	1,362	1,287	1,222	761	885	969	1,073	833	23,302
Annual total	4,283	4,826	6,753	6,983	8,092	7,630	7,303	6,619	7,235	6,972	7,127	6,956	6,635	6,190	6,008	4,259	4,075	3,623	4,081	3,525	119,175

Data Source: (18).

### 3.2 Patients with HD classified based on the degree of disability

**Figure 1:**
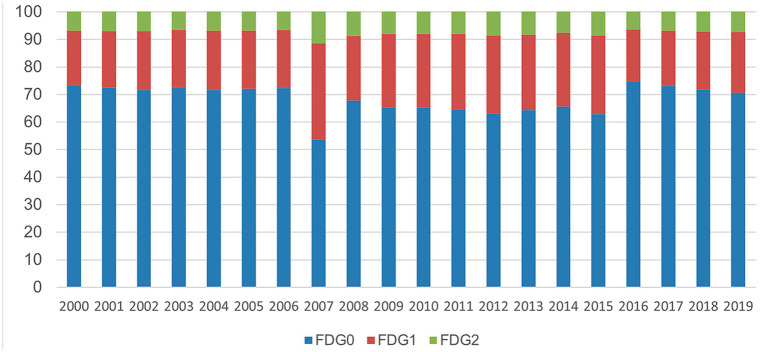
Percentage distribution of patients with HD according to the FDG classification, 2000–2019.

Figure 1 shows the distribution of new HD cases classified according to the Functional Disability Grade (FDG). Higher grades (especially grade 2) indicate more severe cases. This indicator was used to represent the profile of patients with HD who are potentially eligible for lifetime benefits.

The data show an increasing trend in FDG2 cases (green segment of the bars) in recent years. Although this group represents the smallest fraction in the functional classification among the new cases diagnosed each year, it increased by 7.3% between 2000 and 2019. The number of FDG1 cases (red bar segment) increased by 11.6%, whereas the percentage of patients with FDG0 (blue bar segment) decreased by 3.7% in 2019.

The bars in Figure 1 do not show a consistent trend in case severity over time. However, during the last 4 years, a gradual decline in less severe cases (FDG0) has been observed, accompanied by a systematic increase in more severe cases.

### 3.3 Percentage distribution of patients with HD, according to FDG classification, 2000–2019

The FDG percentage assessment column showed a 2.4% increase in the total number of assessments performed at the time of diagnosis. Similarly, the results showed a 0.5% increase in the FDG2 percentage, except for the atypical behavior observed for both variables in 2007 (Table 1).

Although the number of newly diagnosed cases has been gradually decreasing over the years analyzed, which is reflected in the detection coefficient and prevalence indicators of HD (Table 1), the average percentage of FDG2 from 2000 to 2009 was 6.62%, whereas from 2010 to 2019, it increased to 7.9%, indicating that late diagnosis worsened. Consequently, this may directly contribute to the persistence of an epidemiologically hidden endemic and may also imply increased public financial and social support expenditures.

### 3.4 Benefits granted annually in Brazil by the HD from 2000 to 2019

#### 3.4.1 Classifications of benefits granted according to ICD-10

The Social Security database includes the ICD-10 classification for the different stages of HD, with the understanding that this information should serve to justify the granting of the benefit based on its impact on the individual’s daily life and independence. Although the classification allows for some level of detail regarding disease involvement, only one of them clearly expresses the disability acquired by those affected: B92—Sequelae of leprosy, which during this period corresponded to 23,302 benefits granted (19.5%) and represented only 3.1% of new cases of HD from 2000 to 2019 (Table 2).

In contrast, 68,723 (57.6%) of the benefits presented non-specific classifications, such as A30—Leprosy, A30.8—Other Forms of Leprosy, and A30.9—Unspecified Leprosy, in addition to 2,977 (2.5%) benefits identified with the clinical form A30.0—Indeterminate Leprosy. Therefore, 60% of the benefits granted lacked a clear justification or motivation for granting; these classifications are known to represent less severe forms of the disease and do not necessarily lead to significant limitations or disability. Within these broad or undefined typologies, 14,886 (12.5%) beneficiaries received one of the following benefit modalities: a disability benefit under the Continuous Cash Benefit Program (Portuguese acronym: BPC) or Permanent Disability Pension, both of which are lifelong benefits (Table 2).

#### 3.4.2 Benefits classified by modalities of concession

**Figure 2:**
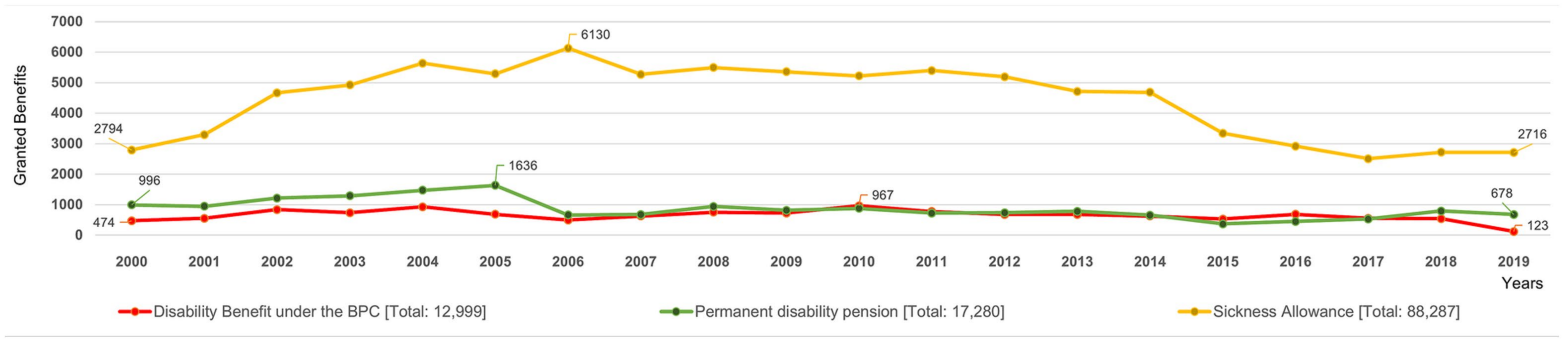
Distribution of the number of benefits granted per species, 2000–2019.

Figure 2 shows three distinct curves, each corresponding to one of the analyzed modalities. Each curve is composed of annual concession totals calculated over 20 years (2000–2019). According to the degree of patient disability, the INSS classifies these concessions into different types of benefits. The most frequent were the disability benefit under the BPC (Continuous Cash Benefit Program, red), the permanent disability pension (green), and the sickness benefit (yellow), totaling 12,999, 17,280, and 88,287 grants, respectively, during the study period.

The temporal behavior of the benefits per species showed overall stability over the analyzed period, especially from 2006 onward, but two opposite inflections were observed in the previous year. First, permanent disability pensions showed an upward trend until 2005, followed by a 59.4% decrease in the following year. At the same time, the disability benefit increased by 46% between 2004 and 2006, and again in 2010, surpassing the number of permanent disability pensions and remaining at this level until the end of the period (Figure 2).

The second inflection refers to sickness allowance, which showed a tendency to increase concessions between 2000 and 2006. It should be noted that between 2005 and 2006, there was a marked 15.8% increase, after which the trend reversed and decreased gently until 2014. From that point onward, the decline became more pronounced, reaching levels similar to those observed in 2000 (Figure 2).

It is noted that the behavior of the curves for disability benefits and permanent disability pensions is opposite to that of sickness allowances. Whereas the first two together accounted for 30.7% of the concessions in 2005, in 2006, both fell to 15.9% of this total. In contrast, the sickness allowance, which totaled 69.3% in 2005, increased to 83.9% of the benefits granted in 2006 (Figure 2).

Considering the epidemiological context of this time interval, Table 1 shows a 12% decline in the diagnosis of new cases (second column) between 2005 and 2006, a substantial percentage for chronic diseases such as HD, characterized by a long incubation period. In the same period, there was an abrupt reduction in the number of permanent disability pension concessions (61.9%) and a 15.8% increase in sickness allowance benefits, as shown in Figure 2. Between these 2 years, the strong variation suggests that changes in the epidemiological context correlate with changes in benefit concessions by the INSS.

In fact, given the decline in the epidemiological picture, a general reduction in the granting of benefits was expected. However, this behavior has occurred more notably only in temporary benefits. This is more evident from 2014 onward, when the granting of sickness allowance was reduced by 42%.

#### 3.4.3 Age and gender distribution of HD beneficiaries, 2000–2019

**Figure 3:**
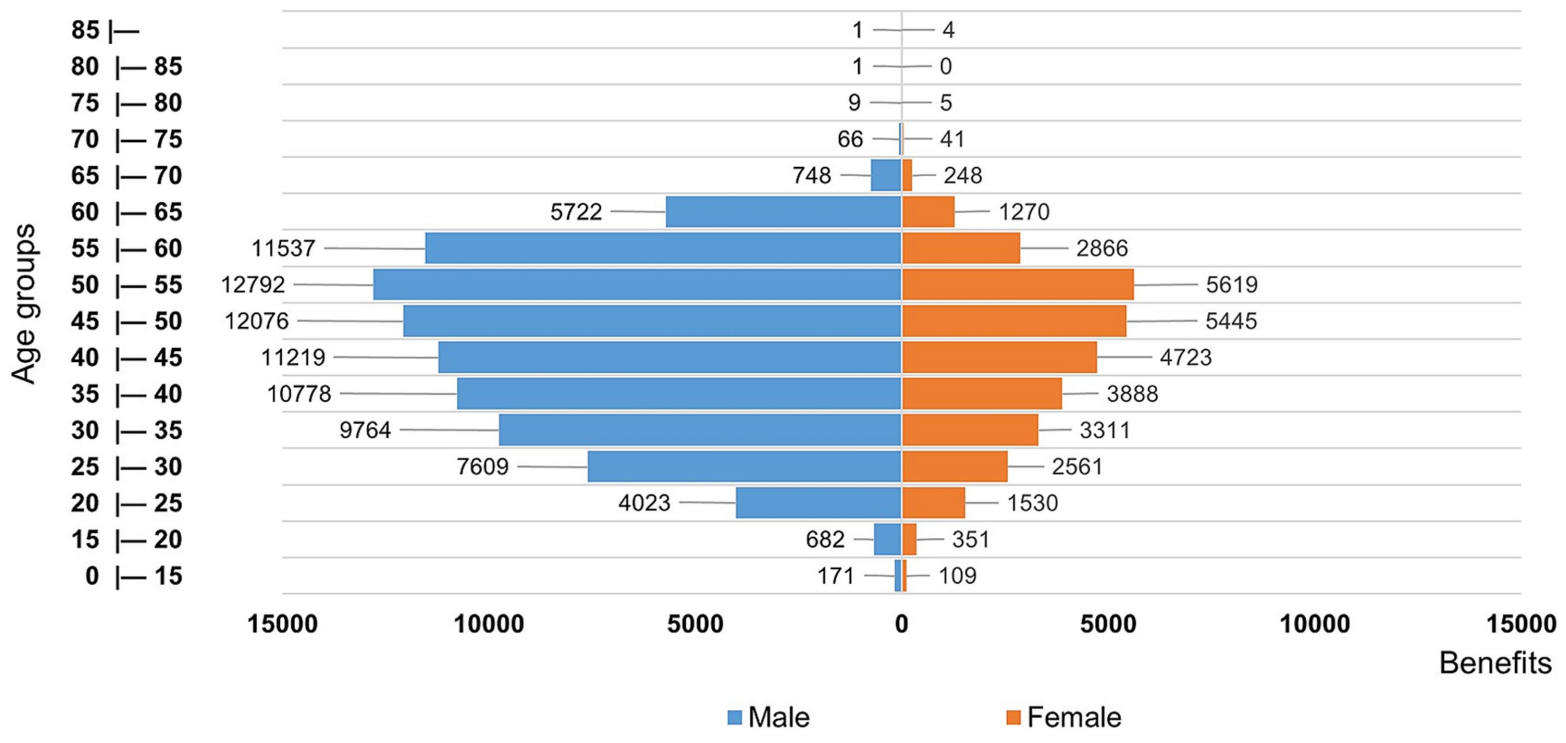
Distribution of age and sex of beneficiaries with HD, 2000–2019. Ages were calculated on the date of benefit provision.

Figure 3 shows the distribution of benefits granted, aggregated by age group and disaggregated by sex. Between 2000 and 2019, a total of 87,198 benefits were granted to men. On average, across all age groups, this number was 2.3 times higher than the benefits granted to women. Overall, considering the 119,175 benefits analyzed, 26.8% were granted to female patients, corresponding to a ratio of 1:2.72, meaning that for every woman who received benefits, nearly three men obtained social security benefits related to HD.

However, no disparity between the sexes was observed in the age at entry into the social security system. In both groups, benefit requests were predominantly concentrated between the ages of 45 and 55 years. The age distributions of men and women were similar, with the number of requests increasing progressively up to the 50–55 age group, after which they declined sharply. Moreover, it can be inferred that a relatively significant contingent of male beneficiaries remained in the 55–60 age range (Figure 3).

#### 3.4.4 Duration of the benefits related to HD

Another topic of this analysis concerns the timings of beneficiaries’ entry and exit from the social security system, an important component in understanding the duration of benefits. It should be noted that a benefit has a duration measured from the granting date to its termination, whether at the request of the beneficiary, medical discharge (cure), or death, among other possibilities.

Of the benefits analyzed, 61,167 (51.3%) lasted up to 18 months. Considering this value, 98.6% were temporary benefits, as reported in the fifth column, indicating that these beneficiaries entered the system with an expected end date or with a scheduled reassessment of their health status (Table 3).

**Table 3:** Duration of benefits related to HD, disaggregated by time and gender during the period 2000 to 2019.

Benefits duration (months)	Frequency							
	Granted benefits	Ceased benefits	Lifelong benefits	Temporary benefits	Gross female	Gross male	*Cum*. female (%)	*Cum*. male (%)
0–6	30,344	30,327	471	29,873	8,577	21,767	26.8	25.0
6–12	19,699	19,554	436	19,263	5,299	14,400	43.4	41.5
12–18	11,557	11,286	370	11,187	2,973	8,584	52.7	51.3
18–24	7,489	7,264	381	7,108	1,911	5,578	58.7	57.7
24–36	10,150	8,926	1,493	8,657	2,541	7,609	66.6	66.4
36–48	6,482	4,919	1,826	4,656	1,672	4,810	71.9	72.0
48–60	4,267	3,077	1,611	2,656	1,113	3,153	75.3	75.6
60–72	3,216	2,066	1,577	1,639	813	2,403	77.9	78.3
72–84	2,453	1,517	1,364	1,089	647	1,806	79.9	80.4
84–96	2,373	1,166	1,588	785	630	1,743	81.9	82.4
96–108	2,143	947	1,603	540	596	1,547	83.7	84.2
108–120	1,954	799	1,560	394	513	1,441	85.3	85.8
120–	17,048	3,416	16,134	914	4,686	12,357	100	100
Grand total	119,175	95,264	30,414	88,761	31,971	87,198		

Data Source: (18). Cum, cumulative.

In turn, the lifetime benefits, represented by the Permanent Disability Pension and the Disability Benefit under the BPC, totaled 30,414, corresponding to 25.5% of all benefits granted in the period. Slightly more than half (16,134) had a duration of more than 120 months (10 years). Considering all HD-related benefits granted in the period, it should be noted that a significant fraction was terminated between 2000 and 2019. By 2019, 85.0% of all benefits had been terminated, of which 64.2% lasted up to 18 months; extending the analysis window to 60 months, 89.6% of benefits were terminated (Table 3).

A breakdown by sex reveals that the columns Gross Frequency of Men and Gross Frequency of Women in Table 3 confirm the predominance of concessions to men, indicating that across all duration categories, the number of male beneficiaries exceeded that of female beneficiaries. In the “Accumulated Percentage by Sex” column, the duration of benefits was similar across groups, with more than half of the benefits (52.7 and 52.3%, respectively) ending within 18 months for both men and women.

#### 3.4.5 Spatial distribution of benefits granted and new cases of HD by Brazilian states

**Figure 4:**
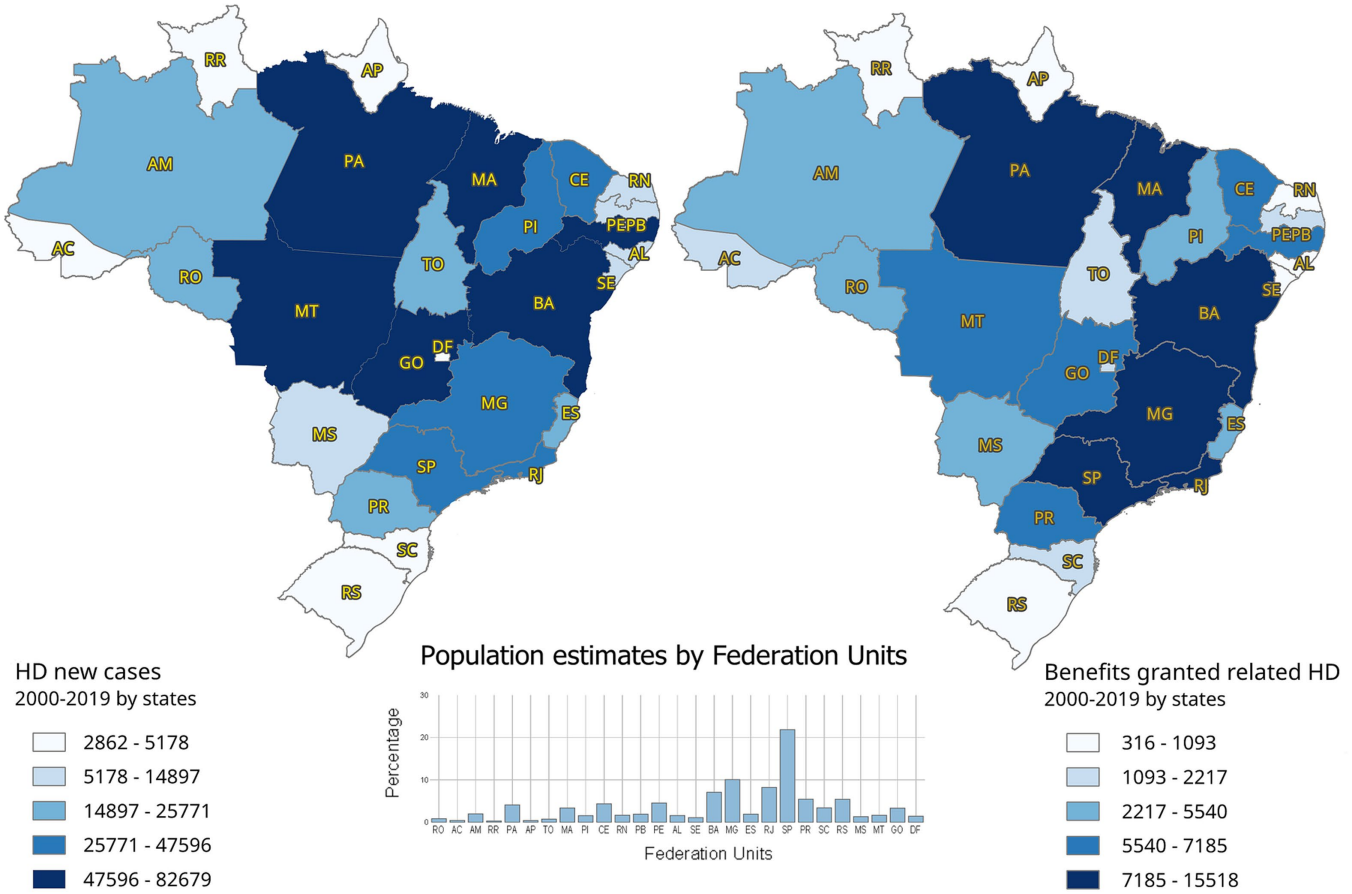
Spatial distribution of new cases of HD and benefits granted by the states, 2000–2019.

Cross-referencing of administrative Social Security data with epidemiological data from the Ministry of Health allowed the recognition of spatial disparities in the granting of benefits throughout the Brazilian territory. The distributions of benefits granted and new cases detected vary widely across Brazilian states and are shown in Figure 4.

To illustrate this, two maps of Brazil are presented side by side. The map on the left shows new cases of HD, classified into five categories by the number of notifications. States in dark blue represent those with the highest incidence, whereas progressively lighter shades indicate lower incidence during the period. The map on the right displays the distribution of benefit concessions related to HD, also grouped into five categories, with states in dark blue corresponding to the highest number of concessions.

Notably, the seven Brazilian states with the highest average percentage of new cases relative to the national total in the period, in descending order, were: Pará (PA, 10.9%), Maranhão (MA, 10.0%), Mato Grosso (MT, 8.6%), Pernambuco (PE, 7.5%), Bahia (BA, 7.0%), Ceará (CE, 5.9%), and Goiás (GO, 5.8%). Together, they accounted for 55.7% of new cases and 47.8% of benefits granted (Figure 4).

However, when the states were ordered by number of benefits granted, Maranhão (MA), São Paulo (SP), Minas Gerais (MG), Pará (PA), Bahia (BA), Rio de Janeiro (RJ), and Mato Grosso (MT) together accounted for 53.4% of new cases and 54.3% of benefits granted. States in the Southeast region (MG, RJ, and SP) accounted for 24.8% of the new cases. However, it concentrated on 21.8% of all benefits granted, indicating that benefit allocation was not directly proportional to disease detection across states.

Figure 4 shows that the states of São Paulo, Rio de Janeiro, and Minas Gerais, among the wealthiest in Brazil, also received the highest number of social security concessions. However, these did not correspond to the states with the greatest number of new cases detected during the period. As shown in the central portion of Figure 4, these states, despite having large populations, recorded fewer diagnosed cases compared with states such as Mato Grosso, Goiás, and Pernambuco, which, even with smaller populations, fell into the highest disease detection category. This reveals a concentration of numerous cases in relatively small populations, whereas a large number of benefit concessions occurred in moderately endemic states.

#### 3.4.6 Public spending on HD-related benefits

**Figure 5:**
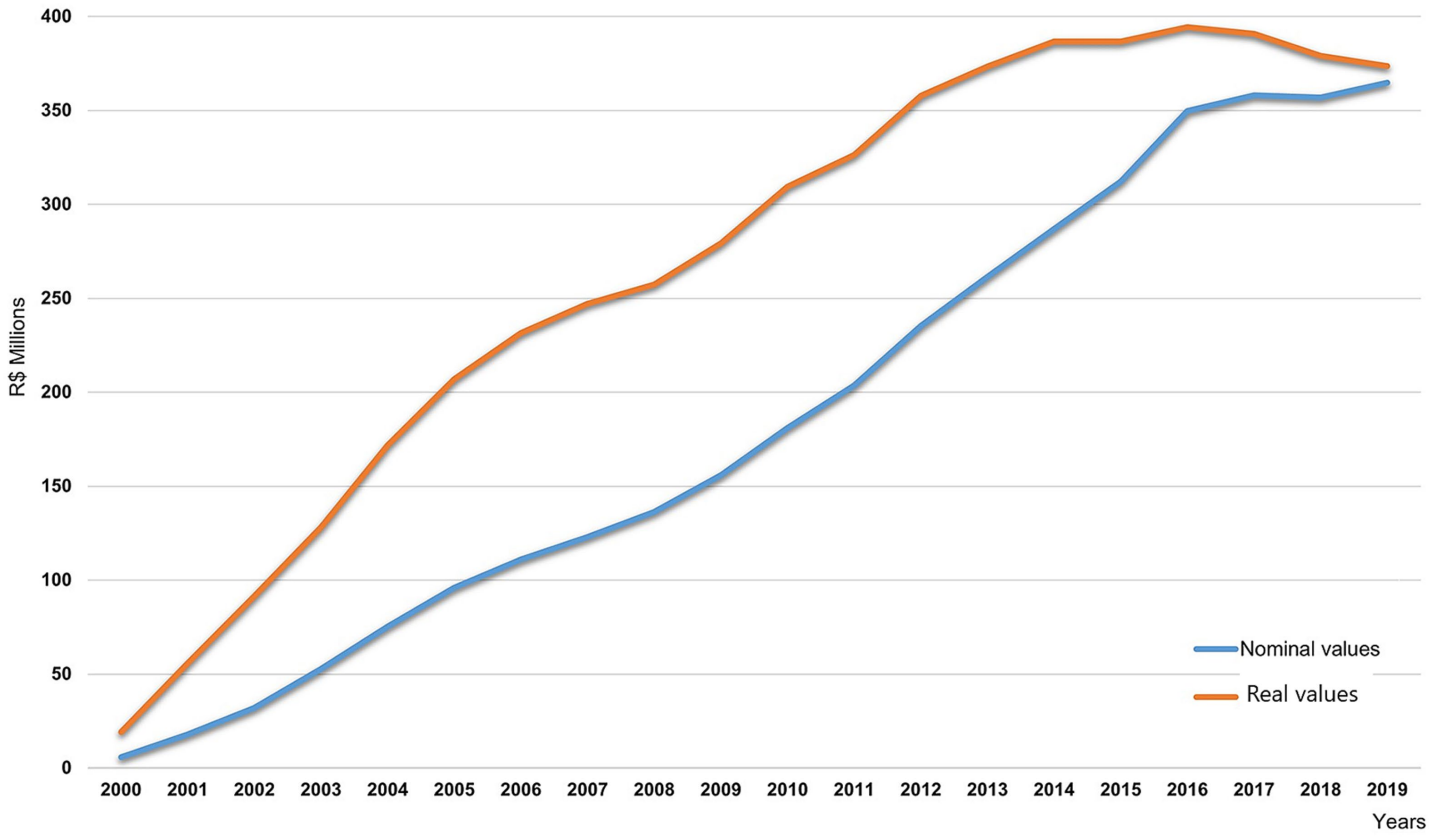
Annual amounts paid to beneficiaries with HD between 2000 and 2019 (real and nominal values). The 13th salary and other additional amounts were not included in the monthly payments.

Figure 5 shows the nominal values (blue curve), which represent the amounts actually paid each year without monetary adjustment, in local currency (Real—R$), alongside the real values (orange curve), corrected to December 2019 to allow for temporal comparability. After adjustment, the real values remain above the nominal curve throughout the period, reflecting the Brazilian currency’s loss of purchasing power and the successive monetary measures implemented to restore its value. This correction demonstrates that the increase in expenditures persisted even after accounting for inflation, reinforcing the sustained economic burden of HD on the social security system.

Public spending on benefits is the most important dimension of the cost of the disease and affects the social security system. Throughout the analyzed period, more than 5.2 million payments were made for HD-related benefits, which totaled US$ 924.6 million (about R$ 4.3 billion in December 2019 values) and approximately R$ 5.2 billion when updated to 2025. In 2000, about R$ 5.7 million (equivalent to US$ 1.5 million) was paid, and in 2019, about R$ 3.7 billion (US$ 90.8 million) was paid, representing an increase of more than 60-fold. The trajectory of the curve indicates that expenditures grew relatively steadily over the period.

## 4 Discussion

This study highlights the substantial economic burden of HD on Brazil’s social security system, which is driven primarily by disability compensation rather than by investments in prevention. By descriptively linking epidemiological and financial-administrative data, it identifies the main affected groups, temporal trends, and regional disparities. These findings reinforce the need for public health strategies that focus on early diagnosis, prevention of disability, and disease control to reduce human suffering and public spending.

An estimated 1.3 billion people worldwide experience significant disabilities caused by HD, representing 16% of the global population or 1 in 6 individuals (22). Physical disabilities due to late diagnosis or insufficient treatment remain a persistent public health problem and are associated with poor socioeconomic outcomes (23).

During the study period, the benefit coverage relative to newly diagnosed cases was 15%. This proportion may suggest underutilization of entitlements or indicate that only a fraction of patients truly require financial support. If the relative proportion of FDG2 is considered in relation to the need for benefits stricto sensu, the effective coverage rate may be even lower. This is because, on average, the relative FDG2 does not reach 10% of newly diagnosed cases within the period.

Among all the benefits granted, 61.45% were classified under generic or non-specific diagnostic categories (A30-Leprosy, A30.8-Other Forms of Leprosy, A30.9-Unspecified Leprosy, and A30.0-Indeterminate Leprosy), limiting the ability to associate benefit concessions with severe disease directly. Of the 71,700 benefits in these categories, 12.5% (14,886) were lifetime benefits (permanent disability pensions or BPC). The absence of clear evidence of disability severity raises concerns regarding the appropriateness of definitive retirement in some cases.

Although HD is included in Brazilian legislation as a condition exempt from the social security grace period (24), the severity of functional limitations must be explicitly determined by INSS medical expertise and correctly classified to ensure the concession of lifetime benefits.

From an epidemiological perspective, between 2005 and 2006, there was an abrupt 11.7% decrease in newly detected cases, with about 6,000 fewer cases in just 1 year, which was attributed to the administrative cleaning of the database. Subsequently, a gradual downward trend continued until 2015. The most severe leprosy cases (FDG2) showed fluctuations over the period but remained consistently around 7% of the newly detected cases.

A parallel assessment of epidemiological trends and benefit concessions revealed a correlation; as cases increased early in the series (2000–2005), benefit concessions increased, and concessions decreased when the incidence declined. During 2005–2006, temporary benefits rose markedly, whereas permanent benefits fell by similar proportions, which may suggest a substitution effect, although causal mechanisms could not be assessed directly in this descriptive analysis.

Detailing this point of view further, in 2005 and 2006, temporary benefits (sickness allowance) increased at proportions similar to those obtained with the reduction in the number of permanent benefits granted (permanent disability pensions and disability benefits under the BPC). Whereas the latter fell to approximately 15% of the total benefits granted, the sickness allowance increased from 69.3% in 2005 to 83.9% in the following year, which corresponded to an increase of approximately 15%, indicating some exchange relationship between temporary and permanent benefits.

At this point, it is worth clarifying the administrative restructuring of the INSS. The INSS medical assessment process has been transformed due to the shortage of doctors and challenges in managing external expert evaluation process. Until 2006, the INSS used accredited doctors, and the agency was responsible only for approving reports by the institute’s experts. This protocol was revoked in 2000 by Decree 3668 and later replaced by Law 10876/2004, which created the career of medical expertise within the administrative structure of the INSS and determined the hiring of 3,000 doctors under a public tender regime and established mandatory protocols and procedures defined by the administration of the state agency. At the same time, outsourced accreditation was gradually abandoned until February 2006.

In 2005, to reduce costs within the system, the Estimated Social Security Coverage (Portuguese acronym: COPES) protocol was implemented, in which the physician defined the discharge period, eliminating intermediate examinations and simplifying the process. If deemed necessary, the beneficiary could request an extension of the concession period to the date of discharge.

In the general context of granting social security benefits in Brazil, from the mid-2000s, benefit concessions have expanded substantially. According to the historical series of the Statistical Yearbook of Social Security-2014, in 2000, the Sickness Allowance totaled 766,888, reaching 1,860,695 in 2005, an increase of 142.6%, whereas permanent disability pensions and disability benefits under the BPC increased by 55.4%. When comparing these types of benefits, both in the general trend and specifically for HD, a disproportionately greater increase in temporary benefits is observed (25, 26).

Another topic of significant divergence among researchers of the Brazilian social security system is the duration of its benefits. Of the 119,175 benefits, 25.5% were lifetime benefits (permanent disability pensions and disability benefits under the BPC). Approximately half lasted for more than 120 months, representing 13.5% of the total benefits granted for HD between 2000 and 2019.

Regarding gender, no significant differences were observed in the duration of the benefit. The cumulative percentages in each gender category were always very similar, often differing in decimal places; i.e., the duration of benefits for men was often equal to that for women. Similarity was also found in the age groups at entry into the social security system (age of request), predominantly between 45 and 55 years for both sexes.

These findings differ from those of previous studies, which argued that women apply for benefits earlier (at a younger age) than men and that their granting lasts longer. Although the benefits of HD retain some specificities that may be biased by broader analyses, the data analyzed do not support the arguments of these authors, revealing no sex differences (26). Despite women forming a smaller subgroup in the sample, no differences in benefit duration were identified between the sexes. Overall, 85% of the concessions were terminated during the study period, of which 51% ended within 18 months, and less than one-third of the lifetime concessions exceeded 10 years.

In contrast, there was a marked disparity between the sexes in the volume of benefits granted, with a male-to-female ratio of 2.7:1, meaning that for each female beneficiary, almost three men received benefits. Although HD affects both sexes, men are more frequently affected, typically at a ratio of 2:1. This male predominance has been documented in several geographical contexts, such as India, the Philippines, Hawaii, Venezuela, and Cameroon, although this pattern is not universally observed (20).

In Brazil, from 2012 to 2016, the disease ratio of the disease between men and women ranged from 1.2 to 1.5 across its regions. However, this epidemiological pattern does not align with the disparity observed in the analysis of social security benefits; a difference of approximately 20 to 50% in favor of men would be expected, and not the nearly 200% identified in this study (20). This disproportion is more plausibly explained by structural gender inequalities in the Brazilian labor market, characterized by higher levels of informal employment among women, lower average wages, and reduced contribution density to the social security system. Consequently, women tend to receive fewer contributory benefits and constitute the majority of BPC beneficiaries (5). The Statistical Yearbook of Social Security 2018 also shows the predominance of benefits granted to men, both in number and total amounts paid. Between 2013 and 2015, an average of 54.5% of permanent disability pensions, disability benefits under the BPC, and sickness allowances were allocated to male beneficiaries, whereas only 39.3% of the total was paid to women during the same period (8).

Inequality was also reflected in the geographic patterns. Geolocated data identified territorial disparities in benefit distribution, with 21.8% of HD-related benefits concentrated in the three states of the southeastern region (MG, RJ, and SP), which together account for 24.8% of newly diagnosed cases and are among the most socioeconomically developed in the country.

This concentration likely reflects the labor market characteristics of the Southeast region, such as higher income levels, population density, and a greater availability of formal employment, which facilitate permanent disability pensions and sickness allowances, which are the most frequent types in this region. In contrast, in areas with a lower supply of formal jobs, applications tend to concentrate on the non-contributory disability benefit under the BPC. Eligibility for this modality is subject to strict social assistance criteria, such as per capita household income below 1/4 of the minimum wage and a fixed benefit amount fixed at one minimum wage (24).

Thus, the availability of financial assistance for patients in some regions of Brazil is limited, which may explain the regional data presented. It is also necessary to consider the contingent of patients who do not fit the legal requirements and have never contributed to social security, as well as those unaware of their rights, who are consequently deprived of the opportunity to receive social security benefits.

Regarding the amounts involved in payments to HD beneficiaries, it was found that there was a significant increase in the amounts disbursed over the period, reaching nearly 60-fold growth, whereas all disability indemnities, permanent disability pensions, and cash assistance for disabled individuals paid by Social Security, regardless of cause, only doubled in the same interval (8, 26). In a broader context, some authors argue that the Brazilian Federal Constitution of 1988 expanded citizens’ rights and, consequently, the state’s obligations, contributing to increased social security expenditures. Even when restricted to HD, the data shown in Figure 5 reflects this upward trend (26, 28, 29).

Although INSS records indicate a gradual reduction in the number of new benefits granted, the total amounts paid continued to increase (Figure 5). This apparent contradiction stems from the legislation in force during the period, which linked the minimum Social Security payment to the national minimum wage. According to researchers, this mechanism contributed to the annual expansion of expenditures on benefits, as each adjustment of the national minimum wage to inflation automatically increased all benefits valued at up to one minimum wage (25–27).

In addition to this readjustment mechanism, the national policy for the appreciation of the minimum wage, which started in 2002 and was consolidated in Law No. 12382 of February 25, 2011, enabled real increases in benefit values each year, resulting in a sustained upward trend of the curves representing the amounts paid between 2000 and 2019.

Benefits of HD experienced a steady increase in the total amount disbursed. Conversely, the reduction in the number of benefits granted, influenced by improvements in administrative processes and declines in epidemiological indicators, was insufficient to counterbalance the rise in expenditures driven by the minimum wage appreciation policy.

The high cost described above becomes even more evident when comparing estimated expenditures with those of other countries. Xiong et al. (30) highlight that improving patients’ quality of life and reducing the economic burden of the disease are essential. Their analysis in a Chinese province showed a median total annual expenditure of US$ 471.00 for migrant patients and US$ 562.90 for residents after diagnosis (30). The study conducted in Ghana by Dalaba et al. (31) reported an average total cost of leprosy treatment of US$ 361.54 per patient, including direct and indirect expenses, and an additional US$ 46.00 for patients with sequelae. Van Veen et al. (32) demonstrated that the average cost of disability prevention per new HD case detected with disability was US$ 44.10, and model-based estimates indicated that preventive interventions cost between US$ 7.00 and US$ 110.00 per day of avoided disability.

In Brazil, no studies have quantified the economic burden of HD, highlighting the need for analyses that assess the impact of chronic diseases on the national economy and support early interventions to reduce long-term governmental expenditure on rehabilitation and retirement. However, using the updated total amount spent from 2000 to 2019, which totaled US$ 924,600,000 for payments to 119,175 individuals affected by HD, an approximate average cost per INSS beneficiary was estimated at US$ 7758.34, considering only the social security benefits paid.

Although the thesis that HD in Brazil is costly is supported, this study has some limitations. From a methodological point of view, as this was a longitudinal study restricted to concessions between 2000 and 2019, neither the benefits previously granted and still active during this interval were included, nor were those granted during the pandemic.

From a conceptual perspective, selecting a single disease with specific characteristics limits inferences to the context of HD, and extrapolations to other health conditions are not recommended. However, this study offers a cross-sectoral analysis between health and the economy, with potential applications in policy formulation, evaluation, and social oversight in the health sector.

Another limitation is that the study did not allow for general conclusions regarding the overall cost of the disease, as only expenditures on social security benefits linked to the INSS were considered. Costs related to routine patient care, management of complications or reactive episodes, rehabilitation demands, amputations, and other clinical needs were excluded. In addition, subsidies and benefits paid by state and municipal pension systems, as well as indemnities guaranteed by Law No. 11520 of September 18, 2007, to individuals historically segregated in former leper colonies were also not analyzed. Another methodological limitation is the absence of data on other forms of retirement, such as pensions paid by federal, state, and municipal social security systems for public servants, which also represent substantial expenditures and could expand the understanding of the disabling impact of HD on the workforce.

From a methodological perspective, it is important to clarify that the use of social security benefit payments as proxies for indirect costs is consistent with a human capital approach, which values productivity losses as the income forgone due to illness or disability. However, this framework may overestimate societal productivity losses, as it assumes that affected workers are not replaced and that all compensated time away from work represents a net loss to the economy. An alternative perspective is offered by the friction cost method, which limits productivity losses to the period required for labor market adjustment and worker replacement, after which production is assumed to resume. Although the friction cost approach may underestimate the broader social consequences of chronic disability, particularly in vulnerable populations, acknowledging this distinction is essential to interpret the present estimates primarily as a measure of the fiscal burden on the social security system rather than as precise estimates of total economic production loss. Future studies incorporating labor market dynamics could further refine the estimates of the economic impact of Hansen’s disease.

The analysis of the impact of HD on public finances helps frame two complementary policy-oriented approaches in the Brazilian context. The first, related to disease control, focuses on reducing the disease burden through prophylaxis, early diagnosis, and improved care for affected individuals, with the potential to reduce the long-term demand for social security resources. The second, of an economic and administrative nature, concerns the organization and management of benefit-granting processes to improve transparency, ensure equitable access to benefits, and optimize costs within the social security system.

Although the present study does not evaluate the effectiveness of specific interventions, the articulation of these two approaches provides a useful framework for interpreting the findings and informing future public policies that seek to balance public health objectives with social security sustainability, fairness, and equity.

## Data Availability

The raw data supporting the conclusions of this article will be made available by the authors, without undue reservation.
